# Feasibility of an interactive voice response system for monitoring depressive symptoms in a lower-middle income Latin American country

**DOI:** 10.1186/s13033-016-0093-3

**Published:** 2016-09-22

**Authors:** Mary R. Janevic, Amparo C. Aruquipa Yujra, Nicolle Marinec, Juvenal Aguilar, James E. Aikens, Rosa Tarrazona, John D. Piette

**Affiliations:** 1 Center for Managing Chronic Disease, University of Michigan School of Public Health, 1415 Washington Heights, Ann Arbor, MI 48109 USA; 2 Universidad Católica Boliviana, Ave 14 de Septiembre 2, La Paz, 4807 Bolivia; 3 Ann Arbor Department of Veterans Affairs Center for Clinical Management Research, VA Ann Arbor Healthcare System, 2215 Fuller Road, Mail Stop 152, Ann Arbor, MI 48105 USA; 4 Estado Plurinacional de Bolivia Ministerio de Salud, La Paz, Bolivia; 5 School of Medicine, University of Michigan, 1018 Fuller St., Ann Arbor, MI 48104 USA; 6 QUANTICA Organización Profesional para el Avance de la Salud Mental, La Paz, Bolivia

**Keywords:** Depression, Depression self-care support, mHealth, Global mental health

## Abstract

**Background:**

Innovative, scalable solutions are needed to address the vast unmet need for mental health care in low- and middle-income countries (LMICs).

**Methods:**

We conducted a feasibility study of a 14-week automated telephonic interactive voice response (IVR) depression self-care service among Bolivian primary care patients with at least moderately severe depressive symptoms. We analyzed IVR call completion rates, the reliability and validity of IVR-collected data, and participant satisfaction.

**Results:**

Of the 32 participants, the majority were women (78 % or 25/32) and non-indigenous (75 % or 24/32). Participants had moderate depressive symptoms at baseline (PHQ-8 score mean 13.3, SD = 3.5) and reported good or fair general health status (88 % or 28/32). Fifty-four percent of weekly IVR calls (approximately 7 out of 13 active call-weeks) were completed. Neither PHQ-8 scores nor IVR call completion differed significantly by ethnicity, education, self-reported depression diagnosis, self-reported overall health, number of chronic conditions, or health literacy. The reliability for IVR-collected PHQ-8 scores was good (Cronbach’s alpha = 0.83). Virtually every participant (97 %) was “mostly” or “very” satisfied with the program. Many described the program as beneficial for their mood and self-care, albeit limited by some technological difficulties and the lack of human interaction.

**Conclusion:**

Findings suggest that IVR could feasibly be used to provide monitoring and self-care education to depressed patients in Bolivia. An expanded stepped-care service offering contact with lay health workers for more depressed individuals and expanded mHealth content may foster greater patient engagement and enhance its therapeutic value while remaining cost-effective.

*Trial registration* ISRCTN ISRCTN 18403214. Registered 14 September 2016. Retrospectively registered

## Background

Depression is the second greatest contributor to disability worldwide [1]. Along with other mental health disorders, depression accounts for a greater share of global disease burden than HIV/AIDS, tuberculosis, diabetes or transport injuries [2]. Besides impairing daily functioning, depression increases the risk of chronic diseases such as diabetes and heart disease, as well as morbidity and mortality associated with these diseases [3, 4]. The negative effects of depression extend beyond the individual to families and society, where lost productivity and medical treatment incur substantial economic costs [5, 6]. Low- and middle-income countries (LMICs) bear most of the global burden of depression [7, 8]. In these settings, adverse social conditions (e.g., poverty, human rights abuses, gender inequality) increase vulnerability to poor mental health [9, 10]. Moreover, severe shortages and the uneven distribution of mental health professionals make conventional treatments inaccessible to most patients who need them [11].

Although there is a lack of reliable population-based data on mental health disorders in Bolivia [12], Latin America as a whole has above-average disease burden due to depression [1], and Bolivia has fewer than 6 mental health professionals (including 1.06 psychiatrists) per 100,000 people, compared to 26.6 across South America as a whole [7, 12, 13]. Fewer than one-fifth of primary care sites in Bolivia have protocols for the evaluation and treatment of key mental health disorders [12]. However, in recent years, mental health care has received increased attention from the Bolivian government including the implementation of a national plan (*Plan Nacional de Salud Mental* 2009–2015), the goal of which is to increase prevention, early detection, and timely treatment of psychological, neurological, and substance use disorders [12]. SAFCI (*Salud Familiar Comunitaria Intercultural*, or Program for Intercultural Family Health Care in the Community) is the major program for providing primary care throughout the country, and includes mental health promotion in its scope [12, 14].

Generally speaking, however, the health system in Bolivia, as in other LMICs, lacks the human resources needed to provide adequate care, including monitoring and self-management support, to patients with depression in primary care settings [12, 14]. Mobile health (mHealth) tools may help fill this gap, yet mHealth solutions have been largely overlooked in efforts to improve the reach of mental health care in poorer countries [15]. Mobile phones are ubiquitous in LMICs [16–19] and in a recent survey of chronically-ill primary care patients in Bolivia, we learned that 86 % had a mobile phone [20]. WiFi has become widely available in Bolivia with stronger signals in recent years due to a communications satellite that was launched in December 2013 [21]. Because of this new national investment in the telecommunications infrastructure, developing mobile health care models is a high priority for the national government.

Interventions based on mHealth strategies tend to have low marginal costs and can reach patients between face-to-face encounters. Randomized trials demonstrate that mHealth interventions can improve self-care among chronic illness patients and may improve health outcomes in LMICs [22, 23] and elsewhere [24]. Mental health care is particularly well-suited for mHealth applications, given that mental health symptoms can be readily monitored [25], and that mental health treatments can be delivered remotely and anonymously in areas where such treatment is stigmatized [15].

Interactive voice response (IVR) technology can be used to monitor depressed patients and provide basic psychoeducation. More than 50 studies have demonstrated that patients with psychiatric symptoms can provide reliable and valid information via IVR [26]. One review of 17 randomized trials with more than 26,000 patients demonstrated that depression symptom reports obtained via IVR are at least as reliable as those obtained using standard methods [26]. While other communication channels such as text messaging and smartphone apps also have advantages, IVR communication can be used to reach patients who have low health literacy, lack more advanced technology and skills, and who are in areas with limited internet connectivity.

In collaboration with governmental officials and academic investigators in Bolivia, we conducted a 14-week demonstration of an IVR monitoring and self-management assistance service among patients with moderate to severe depressive symptoms. The goals of the present study were to: (1) describe the characteristics of program participants, including current depression self-care practices and depression treatments; (2) assess completion rates of weekly IVR assessments and the patient characteristics associated with these rates; (3) assess the reliability and validity of IVR-collected information about depressive symptoms and overall health; and (4) assess participants’ satisfaction with the IVR service.

## Methods

### Patient eligibility and recruitment

Participants were enrolled between July and October 2014 in three primary care centers in La Paz, Bolivia and its sister city, El Alto. Potential participants were initially identified as part of a 2013 survey of chronic illness care and mobile phone use that was conducted in the same primary care sites [20]. We re-contacted survey respondents in 2014 and invited them to complete a follow-up survey about chronic illness care and mobile phone use. Patients were given an additional option to complete an eligibility screening for two other IVR projects: the current depression study and a study conducted among patients with diabetes or hypertension [27]. Patients eligible for the present study had a PHQ-8 score of 10 or above, indicating at least moderate depression [28]. Participants also had to be between 21 and 80 years of age, have access to a cell or landline telephone, and receive most of their medical care at the clinic where they were recruited. Patients were excluded if they had significant memory problems, significant bipolar symptoms, or a diagnosis of bipolar disorder or schizophrenia.

Eligible patients who agreed to participate completed written consent forms, which a research assistant reviewed out loud with them. These forms described the study purpose and process, and stated that all data collected would be kept confidential and would only be used in the aggregate. All IVR responses were monitored by Bolivian and American research assistants, who followed up with patients who consistently missed calls or had elevated depressive symptoms. Bolivian mental health professionals were available for research assistants to consult with as needed.

#### Intervention

Patients enrolled in the depression study received up to 14 weeks of IVR calls. The content of this depression care management tool has been used successfully in the US [29] and was developed with input from US psychiatrists, psychologists, primary care providers, and experts in mHealth program design and health behavior change. IVR scripts were professionally translated into Spanish and reviewed by Bolivian health professionals and community members for cultural and linguistic appropriateness. The automated calling system made multiple attempts to reach patients at times they indicated were convenient, with the goal of achieving one completed call per week per patient. The system verified the person’s identity and patients’ depressive symptoms were assessed using the PHQ-8 [22]. Patients also were asked about their overall health and changes since the previous week in mental and physical health. Based on patients’ touch-tone responses they received feedback about changes in their depression symptom severity along with brief pre-recorded, tailored advice for self-management. For example, participants whose symptoms were getting worse received the following message, based on behavioral activation theory: [30] *Staying in bed all day is not usually a good idea if you are depressed. It’s important to try to get dressed and out of the house each day, even if you do not feel like it. If you continue to need to stay in bed all day you should call your doctor*. Research staff monitored call completion and contacted patients who failed to complete their first week’s call. Alerts based on changes in symptoms were monitored by research staff and sent to patients’ primary care teams.

### Data collection

Upon enrollment and after informed consent, Spanish-speaking research assistants from the University of Michigan administered baseline surveys to participants to gather data on demographics, mental and physical health and treatments, health behaviors, social support, and health care use. Approximately 1 week after the baseline assessment (mean 7.4 days, range 1–15 days), participants received their first IVR call. The IVR system logged each of the system’s call attempts and completed calls, as well as patients’ touch-tone responses to queries. Follow-up surveys were administered to participants either by telephone or in-person by a research assistant and included closed- and open-ended questions about participants’ satisfaction with the program.

### Measures

#### Sociodemographic characteristics

At baseline, participants reported their age, gender, marital status, educational attainment, and problems with functional health literacy. Patients were classified as being of indigenous ethnicity if they reported speaking an indigenous language at home (typically Aymara or Quechua) at least some of the time.

#### Depression-related variables

Depressive symptoms were measured using the Patient Health Questionnaire (PHQ-8). The Spanish translation of the PHQ-9 (which includes a ninth item about suicidal ideation) was shown to be a valid and reliable measure of depression in rural Honduras [31]. Participants were asked if they use any of the following forms of treatment for depression: antidepressant medication, therapy/counseling, exercise, or a healthy diet. The 3-item Sheehan Disability Scale was used to assess depression-associated functional impairment in the domains of work, social life, and family life [32]. A four-item scale screened for post-traumatic stress disorder [33]. Finally, participants were asked, “In the last 6 months, have you been particularly nervous or anxious?”

#### Health and comorbidities

Using a single-item measure of general health perception, participants were asked to rate their overall health on a 5-point scale (excellent to poor). Participants indicated whether or not they had a physician diagnosis for each of 16 common chronic health conditions.

#### Participant satisfaction questions

At follow-up, participants rated the following (1–4, low–high): overall satisfaction with program, perceived quality of the program, likelihood of recommending the program to a friend, likelihood of participating in the program again if offered; and the extent to which the program met their needs and helped them deal with depression. Participants were also asked to describe: the thing they liked best about their experience; what they liked least; and what they would change.

### Data analysis

Descriptive statistics were calculated for demographic, health, and depression-related characteristics of program participants. We calculated the proportion of completed weekly calls out of the total number of active call-weeks, and used one-way analysis of variance (ANOVA) to determine whether this proportion varied significantly across groups defined by participants’ age, gender, education, indigenous ethnicity, overall health, and baseline PHQ score.

We used Cronbach’s alpha to assess the reliability of the PHQ-8 administered during IVR calls. We assessed the construct validity of IVR-reported data on depressive symptoms and self-rated health in two ways. First, we sought to determine whether the information patients reported about their symptoms via IVR was consistent with what they told research assistants in face-to-face interviews at baseline. Specifically, we created cross-tabulations to identify the proportion of participants who reported good or better vs. fair/poor overall health in the baseline survey that also fell in these same two categories based on data from the first IVR call (which was closest in time to the survey). We then repeated this cross-tabulation using PHQ score categories <15 (indicating mild-moderate depressive symptoms) vs. ≥15 (severe symptoms) from baseline survey and first call. Next, we used a one-way ANOVA to assess differences in mean self-rated health across completed IVR calls between groups reporting good/better versus fair/poor health at baseline. We repeated this analysis for mean PHQ-8 score across IVR calls and groups with baseline levels of depression that were severe or less than severe, i.e., PHQ-8 <15 vs. ≥15. Finally, we calculated descriptive statistics for participant satisfaction items and identified dominant themes from open-ended responses.

## Results

### Patient characteristics

A total of 32 patients with PHQ-8 scores of 10 or higher completed the baseline survey and were enrolled in the study (sample characteristics shown in Table 1). Slightly more than half of participants were 45–64 years of age, and about one-third were over 65. Most participants were women (78 %), non-indigenous (only spoke Spanish at home; 75 %), had completed secondary school (63 %) and were able to read (91 %). As might be expected in a sample of chronically ill patients, no participants reported excellent or very good health at baseline; most reported good (31 %) or fair (56 %) health, and almost half (47 %) reported at least five chronic conditions. Diabetes, hypertension, high cholesterol and chronic back pain were all reported by more than 40 % of respondents.

**Table 1:** Baseline characteristics (n = 32)

Variable	Mean (SD) or % (n)
Age	58 years (13)
*Gender*	
Female	78 % (25)
Male	22 % (7)
*Marital status*	
Single	56 % (18)
Married/partnered	44 % (14)
*Education*	
Primary	38 % (12)
Secondary	41 % (13)
>Secondary	22 % (7)
*Language at home*	
Spanish only	75 % (24)
Spanish + Aymara or Quechua	25 % (8)
*Employment* ^*a*^	
Working full-time	44 % (7)
Working part-time	25 % (4)
Homemaker or retired	31 % (5)
*Self-rated health*	
Good	31 % (10)
Fair	56 % (18)
Poor	12 % (4)
*Number of chronic conditions*	
0–4	53 % (17)
5–12	47 % (15)
*Chronic illnesses*	
Hypertension	56 % (18)
High cholesterol	44 % (14)
Diabetes	41 % (13)
Arthritis	28 % (9)
Migraine	28 % (9)
Chronic back pain	47 % (15)
Able to read more than some words	91 % (29)
*High health literacy* ^*b*^	53 % (17)
*Depressive symptoms (PHQ-8 mean)*	13.3 (3.5)
*Sheehan disability scale*	
Work/school life (0–10)	4.1 (2.9)
Social/leisure activities (0–10)	3.9 (2.9)
Family/home life (0–10)	4.0 (3.9)
*Ever diagnosed depression*	34 % (11)
*Current depression treatments*	
Antidepressant use	16 % (5)
Therapy/counseling	6 % (2)
Support groups	6 % (2)
Regular exercise	69 % (22)
Healthy diet	81 % (26)
*Positive PTSD screen* ^*c*^	63 % (20)
*Presence of anxiety* ^*d*^	91 % (29)

^a^16 of 32 respondents were asked about employment status

^b^Rarely or never having problems learning about medical condition because of difficulty understanding written information

^c^As identified by 3 ‘yes’ responses on 4-item screener [33]

^d^Answered affirmatively to “In the last 6 months, have you been particularly anxious or nervous?”

The mean baseline PHQ-8 score was 13.3 (SD = 3.5). Baseline PHQ scores were significantly higher among men than women (means 16.0 vs. 12.7; p = 0.028), but did not differ significantly by ethnicity, education, self-reported depression diagnosis, self-reported health, number of chronic conditions, or health literacy (not shown in Table). Nearly two-thirds (63 %) of participants screened positive for post-traumatic stress disorders (Table 1). On the Sheehan Disability Scale items (0–10 scale), participants reported disruption levels of 4.1, 3.9, and 4.0 for work, social/leisure, and family/home domains, respectively, indicating moderate depression-related impairment (data not included in Table). Almost all participants (91 %) indicated that they had felt “particularly anxious or nervous” in the last 6 months. Few participants reported receiving clinical treatment for depression, although 16 % reported taking anti-depressant medications, 6 % reported receiving individual therapy, and 6 % reported participating in a support group. However, most reported engaging in positive health behaviors for depression self-care; 69 % reported exercising regularly and 81 % reported eating a healthy diet.

### IVR call completion

Patients received IVR calls for an average of 12.8 weeks and completed IVR calls an average of 6.9 weeks, for an overall call completion rate of 54 %. Although there were no statistically significant differences in call completion rate among groups defined by demographic and health characteristics, there was a tendency for higher completion rates among: women (56 vs. 44 % of men), those who graduated from secondary school (60 vs. 44 % of those who did not graduate), participants who spoke Spanish vs. an indigenous language at home (58 vs. 39 %), and those with baseline PHQ scores <15 compared to ≥15 (60 vs. 48 %). (data not shown).

### PHQ and IVR data reliability and validity

**Fig. 1:**
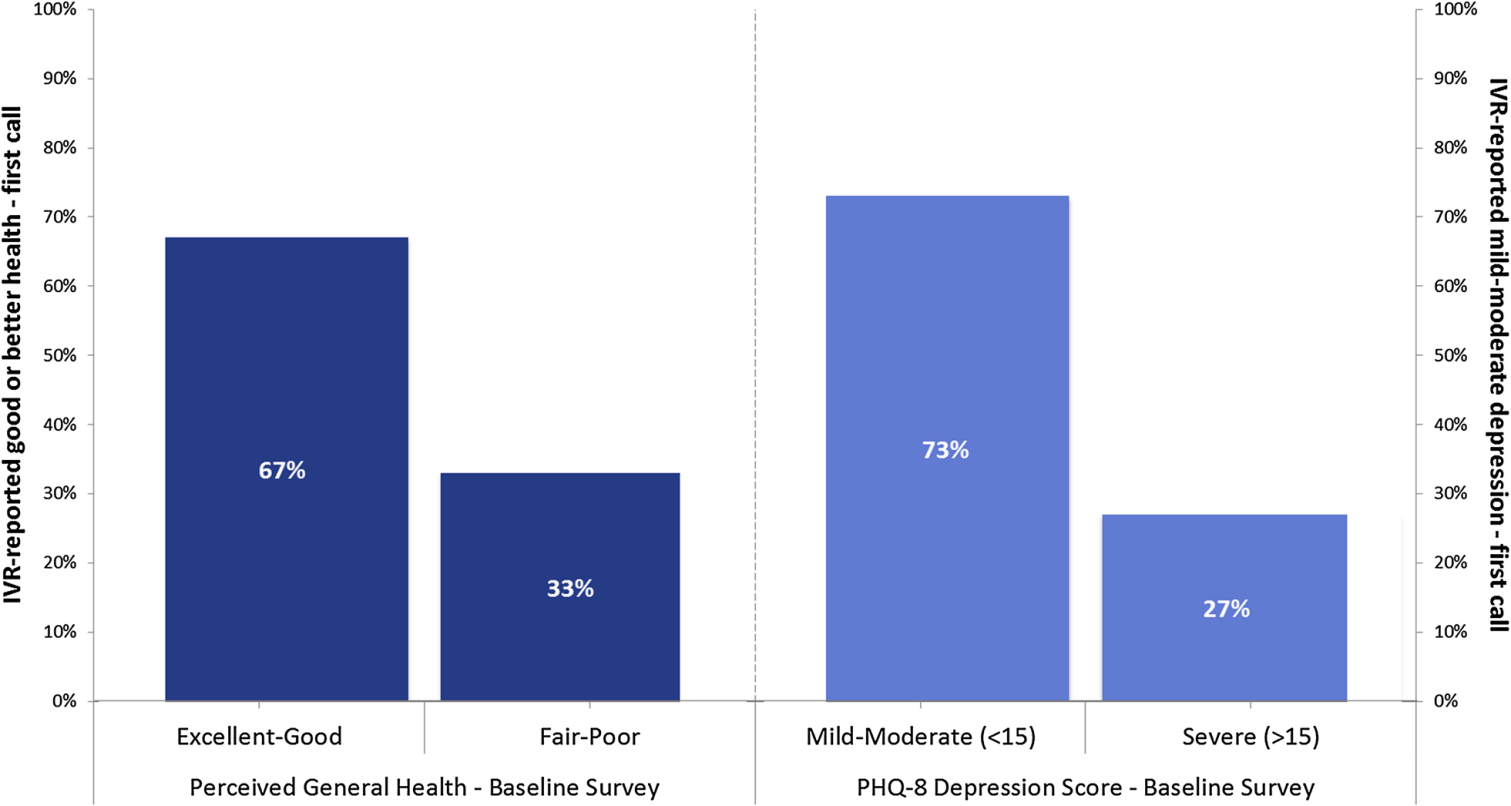
Proportion of patients who reported (*left*, *Y axis*) good or better general health and (*right*, *Y axis*) mild/moderate (< 15) PHQ score on the first IVR call, within groups defined by baseline survey score (*X axis*)

Cronbach’s alpha reliability for PHQ-8 scales completed during the IVR assessments was 0.83. When we examined the bi-variate association between patients’ self-reported health status and depressive symptoms at baseline and what they reported in their first IVR call, the Chi square statistics in both cases were non-significant (p = 0.66 for self-rated health and p = 0.60 for depressive symptoms using Fisher’s exact test). However, as shown in Fig. 1, relationships were in the expected direction: among participants reporting good or better health at baseline, 67 % also reported good or better health “today” during the first IVR call. Among individuals with mild/moderate PHQ-8 scores at baseline, 73 % also reported mild/moderate PHQ-8 scores during the first call. Averaged across calls, mean IVR-reported self-rated health was significantly better among patients reporting good or better health at baseline compared to those with fair or poor health (means 2.4 vs. 3.1; F = 5.5; p = 0.03). The mean IVR-reported PHQ-8 score across calls was likewise lower in the group with mild to moderate baseline PHQ-8 scores, compared to those with moderately severe to severe baseline PHQ-8 depression scores, though the difference did not reach statistical significance (means 8.1 vs. 11.3; F = 1.8, p = 0.19).

### Participant satisfaction with IVR service

Nearly all participants (29/30; 97 %) were “mostly” or “very” satisfied with the overall program, as well as with the amount of help they received. Almost three-quarters of participants (77 %) indicated that the program met “most” or “all” of their needs. All participants reported that they would “generally” or “definitely” recommend the program to a friend. Program quality was rated as “excellent” by 37 % of participants, “good” by 50 %, and “fair” by 13 %. Two-thirds (67 %) indicated that the program helped them “a great deal” with managing their depression. In total, 83 % would “definitely” repeat the program; the remaining participants “probably” would (data not shown in Table).

Table 2 shows major themes and example quotations for each open-ended survey item about program satisfaction. Participants noted a number of beneficial aspects of the IVR calls, including: self-care advice; medication adherence reminders; learning that depression was controllable; having someone ask about their health, the ability of the calls to improve mood; and being able to monitor their depression. Participants also identified aspects of their experience needing improvement, including: repetitive questions, technical difficulties (e.g., dropped calls, problems entering touch-tone responses), timing of calls, and preferring human interaction to a machine.

**Table 2:** Themes from open-ended participant satisfaction items and illustrative responses

Topic/theme	Example responses^a^
*The thing you liked best about your experience*	
Self-care guidance	All the questions are interesting and important because they are concerned about my well-being, telling me how I should take care of myself and how I should take my medications, and get exercise I could see how bad depression could get; I was worried about that and didn’t want to be like that, depressed, and when I listened to the advice I took care of myself
Medication adherence reminders	The reminders to take your medicine as prescribed by the doctor, on the correct schedule, until it becomes a habit (I liked that) they bothered to give reminders about medications, about going to the doctor
Learned that depression can be controlled	I have learned that depression is an illness that you can treat, using the advice that they gave us One learns to trust oneself, go to the doctor, and take control of this illness
Liked having someone ask about health; calls improved mood	It was motivating to have someone call and be concerned about my health, and no matter how sad I was feeling, the advice they gave us always cheered me up Many times they called on a Monday when I was feeling bad, but after the call I felt better
IVR questions helped in monitoring depression	From the ‘how you are feeling’ questions I’ve learned that one can be getting more depressed without realizing it, and the program helped me to realize it, and can look for a good way of thinking what one can do to get out of the situation The advice and the questions taught me how to detect my depression
*The thing you liked least about your experience*	
Questions were repetitious	Sometimes they repeated the same question over and over The last few weeks they kept repeating the same question
Technical difficulties	The calls got cut off a lot and generally I had difficulties in responding—when I was pushing buttons because she said to choose a response, I’d keep pushing but she kept asking the same question again Sometimes the bad thing was that upon typing in my answer, it would hang up and the call was dropped. Maybe a landline would have been better
Calls happened at inconvenient times	I didn’t have any problems with responding, it’s just that often I couldn’t answer the phone because of work or because I forgot my phone at home Sometimes they would call right when I stepped outside
Miscellaneous	I didn’t like that it was a machine, the fact that you couldn’t interact. It’s very “cold.” What I didn’t like is the slant that I felt like the questions had: no matter how good I felt, the program did not pay any attention and when I pressed the response that I felt bad, I was able to continue with the rest of the call It seems very repetitive and long, maybe make shorter calls and later have an interview (in person, like this one)
*If you could change one thing about the service*	
Would not change anything; really liked program	I wouldn’t change anything, all the advice you gave that I could hear I liked Everything was good. I liked how a person that you don’t even know asks you how you are. More advice that tells us to do something or not do something, that helps. Many times I went to the doctor because of the advice that I got
More human contact	I like how the service is now, but I would like to have more personal contact, these would be a lot more helpful than calls (I would like there to be) more personal interviews, to be able to talk to a person and not with a machine

^a^Translated from Spanish. Original-language versions available upon request

## Discussion

Innovative, scalable solutions are needed to address the vast unmet need for mental health care in LMICs. Building on our prior work developing and testing IVR services for chronically ill patients in the United States as well as in Latin American countries, we conducted a feasibility study of an automated telephone interactive voice response (IVR) depression self-care service among 32 Bolivian primary care patients with initially-elevated PHQ-8 scores. Call-completion rates over 14 weeks, as well as internal-consistency reliability and construct validity of IVR self-reported health and depression data were all within acceptable ranges. Based on screening items in the baseline survey, psychiatric comorbidities such as PTSD and anxiety disorders appeared to be highly prevalent in this sample. Few individuals were receiving professional treatment for depression outside of the service, though most reported engaging in depression self-care. Participant satisfaction with the IVR service was generally high, although some described technical challenges and the limitations of receiving IVR-only contact to address depression. Overall however, findings provide some evidence for the feasibility and acceptability of an IVR service to support depression management in this population, and point to ways to modify this service that may foster greater patient engagement and enhance its therapeutic value.

### IVR call completion

Call completion rates were similar to those in other recent studies of patients with diabetes and hypertension in Bolivia [27]. This is encouraging, because it suggests that despite the hopelessness and passivity that often accompany depression, patients were able to engage in this form of self-care support at a level comparable to a broader group of chronically-ill patients. Also, even with an overall call completion rate of only 54 %, this type of intervention represents a level of patient monitoring and psychoeducation that substantially exceeds what patients currently receive through standard outpatient encounters. On the other hand, the call completion rates that we have observed in Bolivia are lower those we have seen in Honduras and Mexico (roughly 65 %) and among Spanish-speakers in the U.S. (roughly 80 %) [34]. Corresponding rates among English-speaking people with depression in the US are roughly 71 %, and other English-speakers with chronic illnesses complete their calls 85–90 % of the time [35]. It is unknown whether call completion rates as low as the one we observed in this present study represent an adequate “dose” of depression self-management support. Given feedback from some participants about technical difficulties with the calls and dissatisfaction with some aspects of call structure, it is possible that addressing these issues would boost call completion rates.

We found that participants who spoke an indigenous language (Aymara or Quechua) at home—a proxy for ethnicity or degree of ethnic affiliation—had substantially lower call completion rates than participants who spoke Spanish only (58 vs. 39 %). Participants who were more depressed and did not complete secondary school also had notably lower call completion rates; though in this small sample, these differences did not reach statistical significance. Future versions of the program should pay particular attention to the needs of these groups, including translation into indigenous languages, and address their barriers to call completion. Finally, in a recently published pilot trial [36], we found that chronic disease patients in Bolivia who are indigenous, or who have low health literacy or poor medication adherence, are more likely to complete IVR calls when they enroll into the program along with a family caregiver who receives regular feedback about their health and call completion. Other studies from the US and other Latin American countries suggest that providing feedback to family caregivers increases patient engagement as well as program impact upon health and self-management behaviors [29, 35, 37–39]. Broadening the current depression self-care support program to include family members may therefore enhance patient engagement and outcomes.

### Reliability and validity of IVR-collected data

The internal consistency reliability (Cronbach’s alpha) of the PHQ-8 collected in IVR calls was good at 0.83, and almost identical to a previous coefficient of 0.84 in an unpublished analysis by the authors of data from an in-person survey delivered in a sample of 600 Bolivian primary care patients. Researchers elsewhere in Andean Latin America have confirmed the scale’s cultural appropriateness [40]. We assessed the validity of IVR-collected self-rated health and depressive symptom data by testing whether it related in expected ways to the survey data, which we assumed was more accurate as it was collected using a well-established standard method. These associations were in the expected directions, although they did not always reach statistical significance. We note that the association between baseline survey- and IVR-collected data of baseline PHQ-8 and self-rated health scores would naturally be attenuated by the fluctuation of depression symptoms over time. The self-care messages included in the calls may also have alleviated depressive symptoms in some cases. Nonetheless, the patterns we observed in our data were consistent with our hypothesis that patients in the Bolivian primary care system with elevated depressive symptoms can accurately report their health and mood status via IVR calls.

### Depression characteristics, treatment and self-care

Over three-quarters of patients in our sample were female. Across countries and cultures, women are more likely to be depressed than men [41, 42]. Notably, the men in our sample had significantly higher baseline PHQ scores, possibly reflecting that women are willing to participate in mental health programs at a lower level of symptomatology. Although the mean overall baseline PHQ score for the sample (13.3) fell near the center of the “moderate” range of depressive symptom severity, only one-third of participants reported having a depression diagnosis. As expected given the scarcity of mental health workers and psychotropic medications in Bolivia, as in other LMICs [10, 43] as well as a weak infrastructure for identifying and treating primary care patients who have mental health disorders [44], depressive symptoms appeared to be virtually untreated in our sample. Very few participants reported taking part in either counseling or support groups for depression, or taking antidepressant medications. In contrast, a large majority reported either exercising regularly or following a healthy diet to self-manage depression. This fact demonstrates a willingness to engage in self-care for depression in the form of positive health behaviors, as observed also in participants’ responses to open-ended questions. Finally, our participants tended to report only low to moderate functional impairment due to depression. Individuals with higher levels of functional impairment may be unlikely to participate in an IVR-based self-care program.

Comorbid mental health disorders appeared to be highly prevalent in our sample, with nearly two-thirds of participants screening positive for post-traumatic stress disorder and over 90 % indicating possible anxiety disorder (i.e., they had been particularly anxious or nervous in the last 6 months). Comorbidity between psychiatric disorders is common in LMICs, and in these settings it is often impractical to administer separate evidence-based interventions for co-occurring psychiatric illnesses [45]. The common elements treatment approach (CETA) developed by Murray and colleagues for delivery by non-specialists incorporates flexible treatment elements targeting a wide range of psychological symptoms. Because CETA involves weekly symptom monitoring to inform treatment element selection and dose, this approach may be well-suited for incorporation into IVR or other mHealth interventions to treat a variety of common, and/or comorbid, mental health disorders.

### Participant satisfaction with the IVR service

Some participants (Table 2) revealed in open-ended comments that they valued the attention, guidance, and feedback that they received as a result of participating in the program. Some comments implied at least a temporary therapeutic effect of the IVR contact; for example: “*It was motivating to have someone call and be concerned about my health, and no matter how sad I was feeling, the advice they gave me always cheered me up*.” The messages regarding self-care and taking control of depression seemed to resonate with participants, even in a culture that is often seen as fatalistic and passive: “*One learns to trust oneself…and take control of this illness*.”

Nonetheless, responses also suggested areas for program improvements. A number of participants felt that the program would be more compelling if it incorporated a “human element.” “Task-shifting” in the form of training lay and non-specialist health workers to deliver brief, structured psychological treatments under specialist supervision is an evidence-based approach recommended by the World Health Organization for delivering mental health care in LMICs [46, 47]. Psychotherapeutic interventions that combine electronically-delivered elements with contact with a non-specialist health worker are growing in use [48] and may be a low-cost way to augment mHealth interventions with personal contact, while at the same time leveraging the structure and consistency of the automated electronic components to increase the capacity of lay health workers to provide high-quality psychotherapy. In Bolivia, a manual for delivering cognitive-behavioral therapy that is based on WHO guidelines for depression care and also tailored to the specific socio-cultural context of Bolivia is under development; this is an important resource that will facilitate training local health workers (Dra. Rosa Tarrazona, personal communication, January 26, 2016).

Technical difficulties during the IVR calls, such as problems entering data or completing their calls, were frustrating to participants. Aside from addressing this problem with improved technology, if available, live technical support would have helped participants resolve problems with their phone or the IVR system, and may reduce program attrition. Last, one participant alluded to need to address the role of intimate partner violence: “[*Maybe there should be] advice for couples, because there are many women who are beaten by their husbands and they get depressed and they need help getting out of this problem*.” (Quotation not shown in Table 2.) This comment, along with data indicating that nearly half of all Bolivian women reported experiencing violence from their intimate partner in the last year [49], point to the importance of addressing social and safety issues as part of the service, including a mechanism for referrals to appropriate resources.

## Limitations

All participants in our sample were Spanish-speaking, whether or not they also spoke an indigenous language at home. Thus, it is unclear whether our program would be feasible or acceptable to monolingual speakers of indigenous languages in Bolivia, who are more likely to live in poverty and lack access to health care than Bolivians who can speak Spanish. Our small pilot sample was recruited from a list of primary care patients who responded to a previous survey about chronic illness care; therefore, the disease burden in our sample was high. However, given the strong link between physical illness and depression, future users of depression-support programs in this setting will almost certainly also have a high degree of physical comorbidity. Finally, this small feasibility study was not designed to assess whether the IVR service alleviates depressive symptoms. Nonetheless, the data collected and lessons learned have been used to inform the development of an expanded version of the program in a larger, ongoing trial being conducted in collaboration with Bolivian academic and government institutions.

## Conclusion

The present study supports the potential of the emerging field of global mental mHealth, in which a highly-prevalent resource (mobile devices) is applied to a highly-prevalent need [15]. However, our findings also draw attention to possible limitations of depression management-support interventions in LMICs that are based on brief, electronic interactions only. Few of our participants were receiving formal depression treatment, and our qualitative data suggested that the program could be improved by augmenting the content and varying it more across weeks, as well as including a human element. Therefore, a significantly expanded program that includes a more comprehensive, interactive IVR element, as well as some degree of interaction with a lay health worker, seems well-justified. Even limited contact with a health worker, and/or a family member who receives automated updates and alerts, may encourage greater engagement with IVR calls. Future testing of programs incorporating these elements will help refine a model for depression treatment that is both effective and sustainable within the under-resourced health care systems of LMICs.

## Abbreviations

LMICs: lower and middle-income countries
IVR: interactive voice response
PHQ: Patient Health Questionnaire
ANOVA: analysis of variance
SD: standard deviation
U.S.: United States
